# Orthognathic surgery for patients with fibrous dysplasia involved with dentition

**DOI:** 10.1186/s40902-018-0176-y

**Published:** 2018-12-03

**Authors:** Santhiya Iswarya Vinothini Udayakumar, Jun-Young Paeng, So-Young Choi, Hong-In Shin, Sung-Tak Lee, Tae-Geon Kwon

**Affiliations:** 1 Department of Oral and Maxillofacial Surgery, School of Dentistry, Kyungpook National University, 2177 Dalgubeol-daero, Jung-gu, Daegu, 41940 Republic of Korea; 2 Department of Oral and Maxillofacial Surgery, Samsung Medical Center, Sungkyunkwan University School of Medicine, Seoul, Republic of Korea; 3 Department of Oral Pathology, School of Dentistry, Institute for Hard Tissue and Bio-tooth Regeneration, Kyungpook National University, 2177 Dalgubeol-daero, Jung-gu, Daegu, 41940 Republic of Korea

**Keywords:** Fibrous dysplasia, Orthognathic surgery, Le fort I, Osteotomy, Rigid fixation

## Abstract

**Background:**

Fibrous dysplasia (FD) is characterized by the replacement of normal bone by abnormal fibro-osseous connective tissue and typically treated with surgical contouring of the dysplastic bone. When dysplastic lesions involve occlusion, not only is surgical debulking needed, orthognathic surgery for correction of dentofacial deformity is mandatory. However, the long-term stability of osteotomized, dysplastic bone segments is a major concern because of insufficient screw-to-bone engagement during surgery and the risk of FD lesion re-growth.

**Case presentation:**

This case report reviewed two patients with non-syndromic FD that presented with maxillary occlusal canting and facial asymmetry. Le Fort I osteotomy with recontouring of the dysplastic zygomaticomaxillary region had been performed. The stability of osseous segments were favorable. However, dysplastic, newly formed bone covered the previous plate fixation site and mild bony expansion was observed, which did not influence the facial profile. Including the current cases, 15 cases of orthognathic surgery for FD with dentition have been reported in the literature.

**Conclusion:**

The results showed that osteotomy did not appear to significantly reduce the long-term stability of the initial fixation insufficiency of the screw to the dysplastic bone. However, based on our results and those of the others, long-term follow-up and monitoring are needed, even in cases where the osteotomized segment shows stable results.

## Background

Fibrous dysplasia (FD) is a benign, developmental, nonheritable, and slowly progressing disorder of the bone characterized by replacement of the normal bone by gradual abnormal proliferation of immature, irregularly distributed fibro-osseous connective tissue. FD is caused by a gene mutation that affects both bone formation and resorption [1, 2]. The skeletal involvement varies from monostotic (single bone) to polyostotic (multiple bones), leading to progressive functional deficits and reduced esthetics. When FD involves only one bone, and not contiguous multiple bones in the skull, the disease is characterized as monostotic rather than polyostotic [3]. Therefore, craniofacial FD without involving other skeleton, such as the femur or rib, would be commonly defined as monostotic FD [4]. The maxilla and frontal bones are the most commonly involved bones in the craniofacial region. The typical appearance of patients with FD of the maxillofacial bone is facial asymmetry caused by a significant expansion of the bone [2]. When the maxilla is involved, an increase in the prominence of cheek is observed. Since the craniofacial FD is not well delineated, conservative surgical debulking is usually performed to restore the facial contour. However, the FD lesion sometimes affects the alveolar bones of the maxilla or mandible, causing discrepancy in the occlusion secondary to the alveolar bone expansion. In such cases, complete osteotomy and repositioning of the maxilla or mandibular bones must be performed concomitantly with debulking of the lesion [5–10].

The main goals of orthognathic surgery for FD are to contour the excessive bone lesion, correct the dentofacial deformities, and restore the occlusion. Because of the frequent obliteration of the maxillary sinus and the anatomical abnormality at the vascular structures, adequate osteotomy is challenging [8]. In addition, the dysplastic nature of the FD lesion cannot afford insufficient screw-to-bone engagement [7]. The major concerns following orthognathic surgery are suitable bone reunion, quality of the newly formed bone, which can potentially influence the stability of the osteotomized segments, and FD recurrence.

The purpose of this study was to investigate the management of FD in cases with various degrees of dentofacial deformity with occlusal discrepancy. Bone healing of osseous segments and the prognosis of orthognathic surgery for FD involved with dentition in the current and previous cases is also reviewed.

## Case presentation

### Case 1

**Fig. 1:**
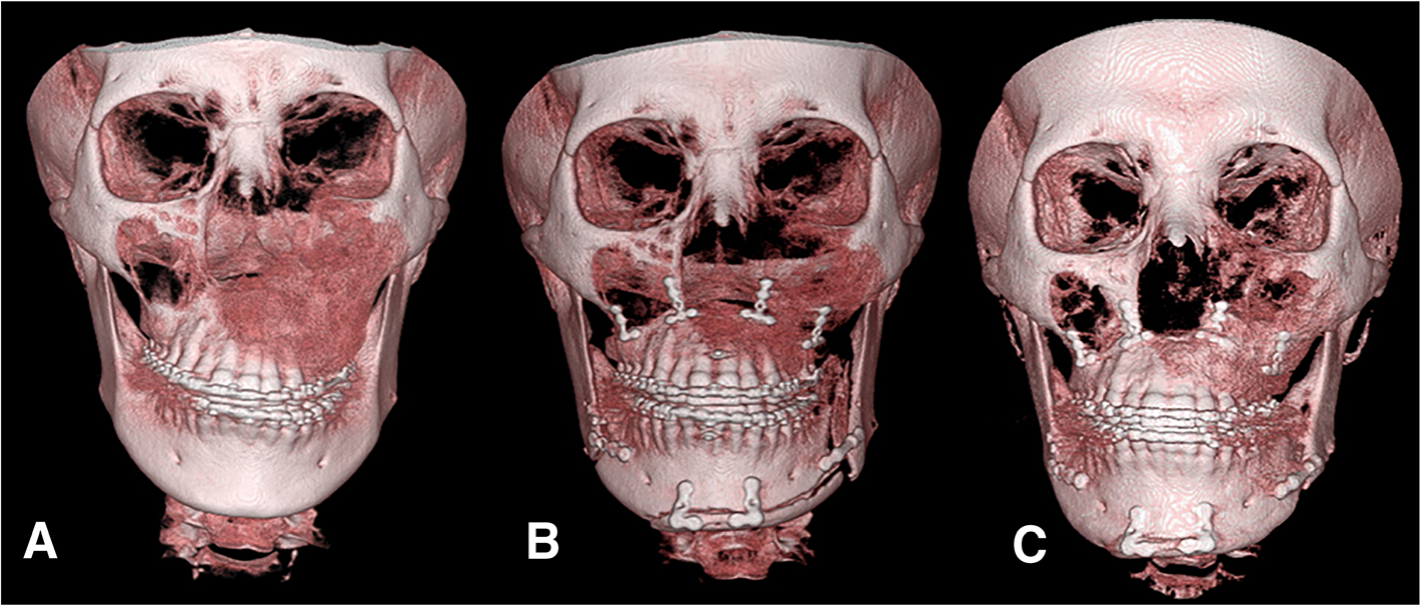
3D CT image of patient no. 1 preoperative (**a**) immediately postoperative (**b**) and 1 year postoperative status (**c**) showing improvement in occlusal canting following Le Fort I and wing osteotomy on the mandibular body and chin with rigid fixation

A 13-year-old girl visited our department with complaints of swelling and facial asymmetry on the left side of the face. The patient was first diagnosed as craniofacial monostotic FD involving the left maxilla, subnasal, and zygoma. The patient underwent primary bone contouring surgery under general anesthesia. At age 18, the patient presented with a swelling on the left side of the mid face region, suggesting lesion re-growth. On clinical extra-oral examination, a significant facial asymmetry was present. Computed tomographic (CT) images showed a typical dysplastic dense mass affecting the maxillary and zygomatic bones. The preoperative cephalometric analysis showed canting of 5.5 mm downwards. After confirming the cessation of the growth, presurgical orthodontic treatment was continued for 14 months. At age 20, final surgical treatment objectives were established. The patient exhibited severe maxillary occlusal canting with gummy smile and compensatory mandibular occlusal canting, but showed a relatively symmetrical outline of the mandible. Le Fort I osteotomy was performed to correct occlusal canting, and the dysplastic bone obliterating the maxillary sinus was also removed. Concomitantly, a massive amount of friable dysplastic bone from the maxillary alveolar bone to the zygoma and infraorbital areas was extensively removed. Mandibular sagittal split ramus osteotomy (BSSRO) was performed to correct compensatory mandibular occlusal canting. To adjust the mandibular chin and body contour, Triaca style mandibular wing osteotomy [11] was also performed. Osteotomized maxilla was rigidly fixed with four 1.5-mm-thick microplates whereas BSSRO was fixed with two 2-mm-thick miniplates (Fig. 1a, b). The fixation of the screws was slightly weaker at the FD-involved side than the contralateral normal maxillary bone.

**Fig. 2:**
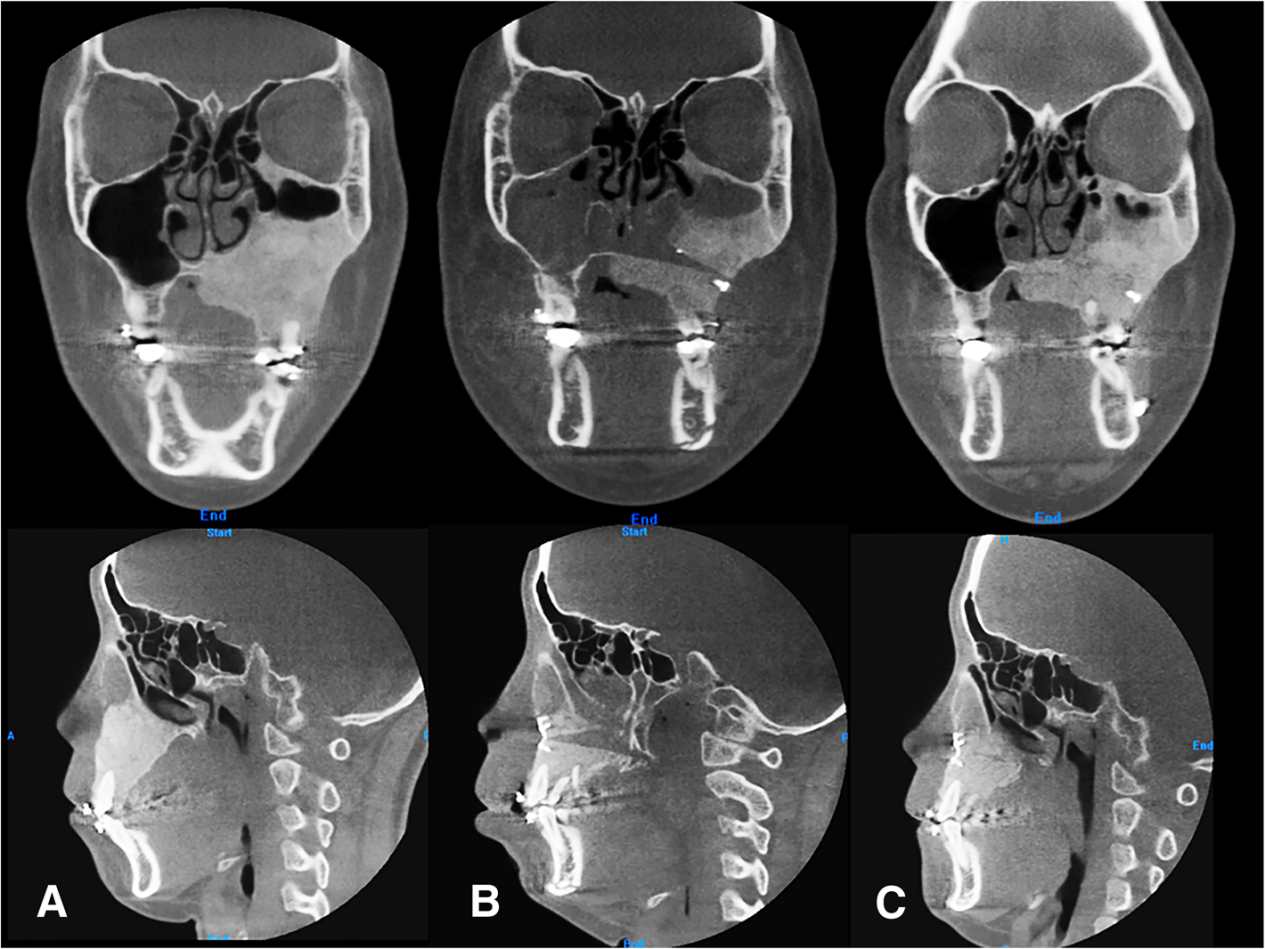
Coronal (upper panel) and sagittal (lower panel) images of patient no. 1 preoperative (**a**), immediately postoperative (**b**), and 2 years postoperative (**c**). Bone union at the osteotomized bone was composed of dysplastic bone, and the maxillary sinus was obliterated again. The plates were covered by the newly formed dysplastic bone

The postoperative course was uneventful, and postoperative orthodontic treatment was started 4 weeks after the surgery. There was no evidence of skeletal relapse at the postoperative 2-year follow-up (Fig. 1c). The coronal and sagittal views of the CT showed that the bone union at the osteotomized bone was composed of dysplastic bone, and the maxillary sinus was obliterated again. The plates were covered by the newly formed dysplastic bone (Fig. 2). However, slightly expanded external cortex of the left zygoma and maxilla on the left side did not influence the facial symmetry. The patient did not want further surgery and was satisfied with the final outcome.

### Case 2

**Fig. 3:**
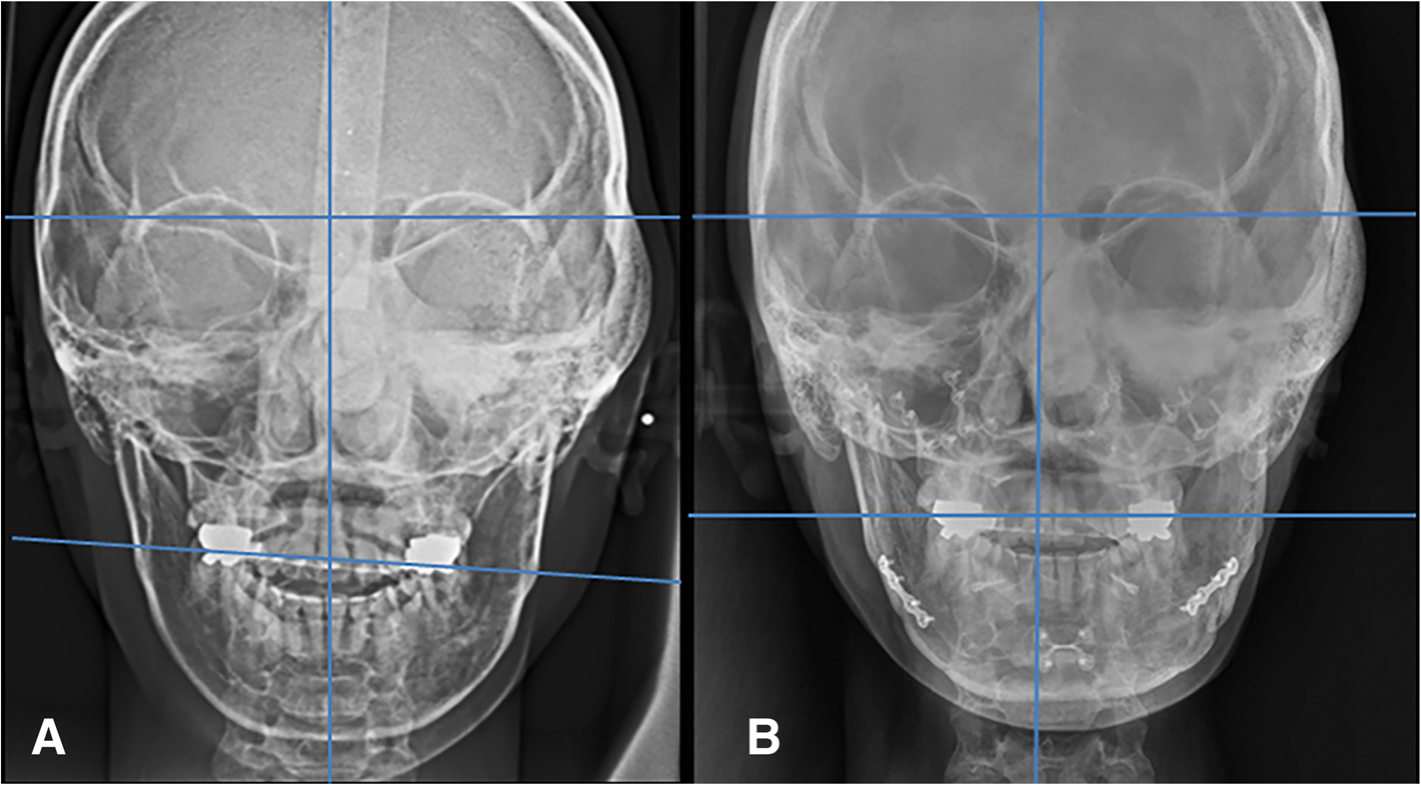
Preoperative (**a**) and 2 years postoperative radiographic image (**b**) of patient no. 2

**Fig. 4:**
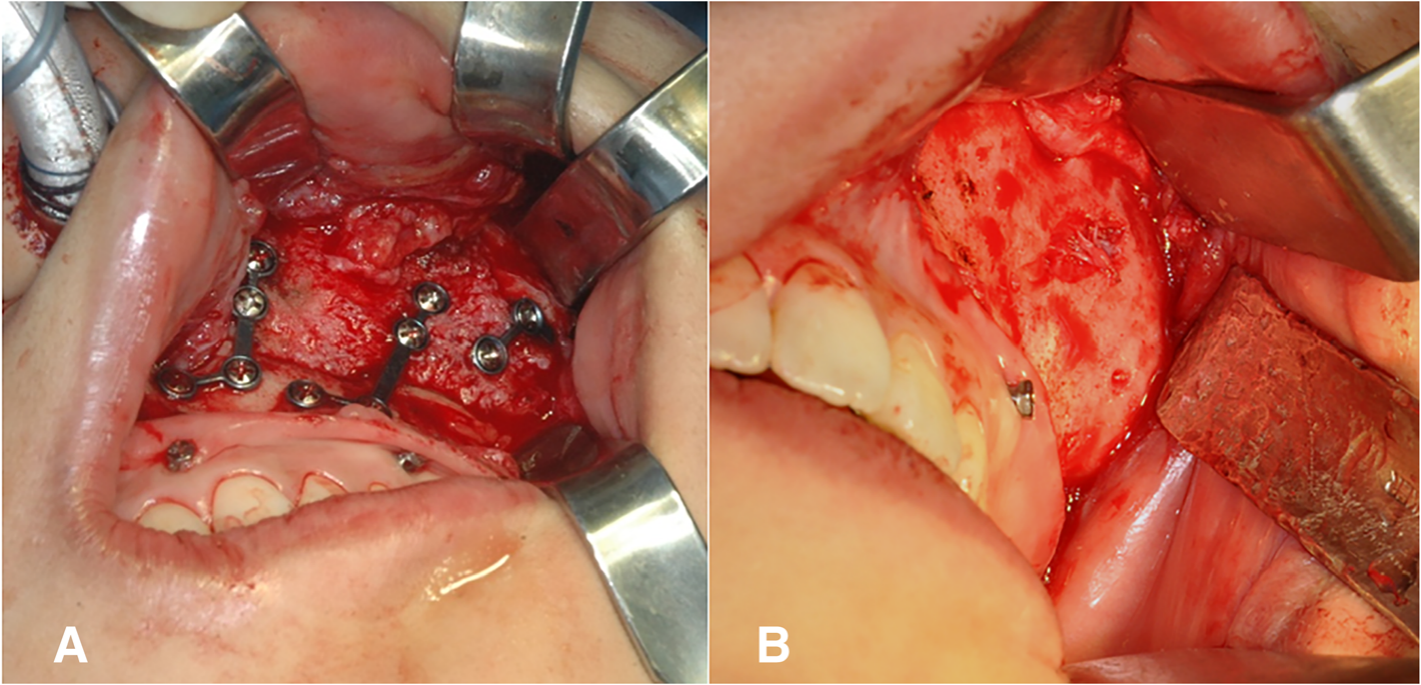
Intraoperative (**a**) time of plate removal at 2 years after the initial surgery (**b**). The previous site of the maxillary Le Fort I osteotomy was revisited, and significant osseous union was noted between the osteotomized segments. Slight expansion of the external cortex of the left maxilla was also noted, which did not affect facial symmetry

**Fig. 5:**
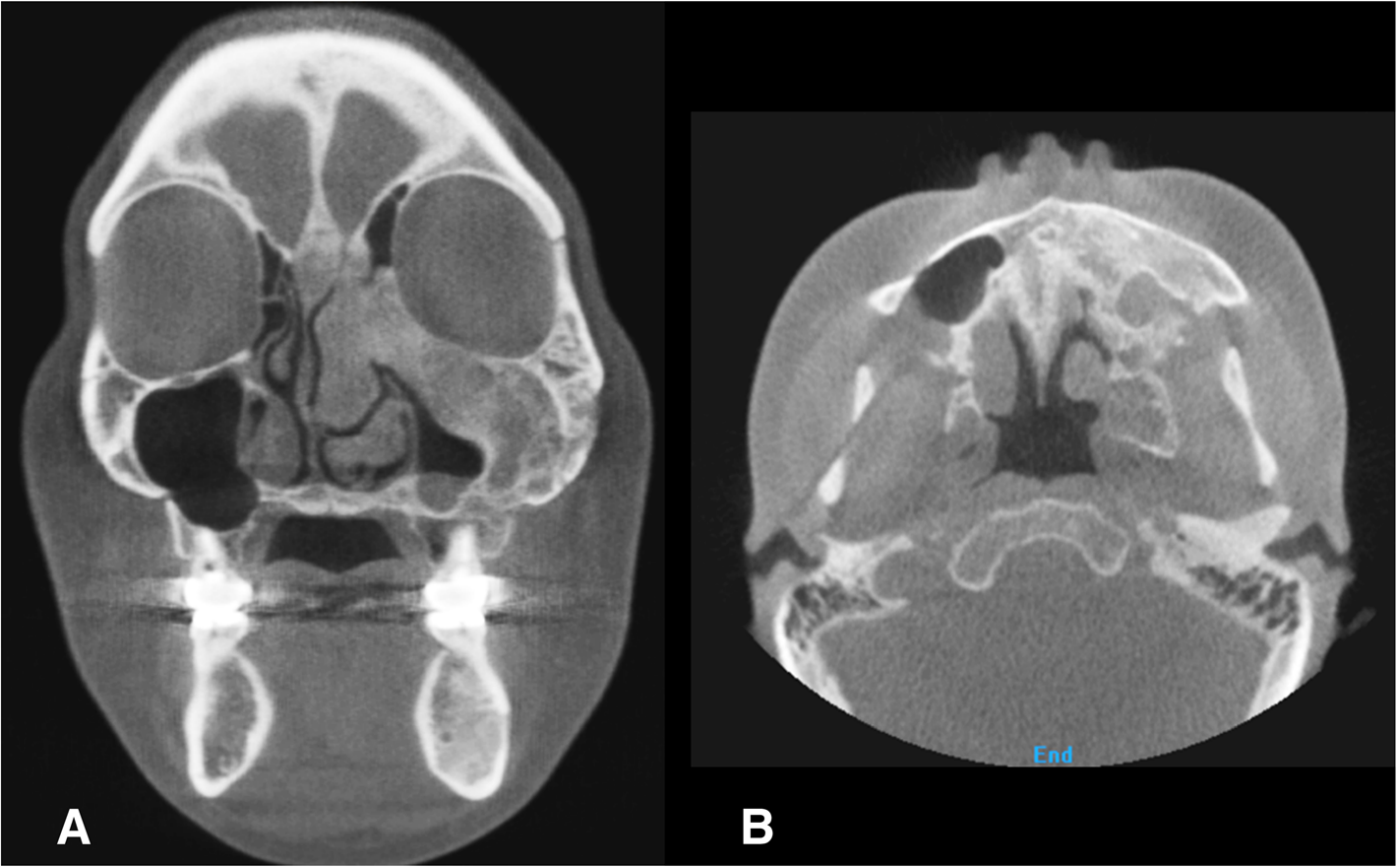
3D CT image of coronal (**a**) and axial (**b**) view 4 years postoperative. No further expansion or re-growth of the dysplastic lesion was noted

A 29-year-old woman complained of swelling on the left side of the face. CT images showed a radiodense network of the expanded dysplastic bone lesion involving maxilla, zygoma, ethmoid bones, orbit, cranial base, and hemimandible. Therefore, the patient was diagnosed as polyostotic craniofacial FD. The patient did not exhibit visual disorders. The preoperative cephalometric analysis showed canting of 5 mm downwards. An orthognathic surgical procedure was planned. Le Fort I osteotomy, sagittal split ramus osteotomy, and genioplasty were performed under general anesthesia (Fig. 3). To ensure maxillary impaction to correct occlusal canting, the FD lesion was extensively removed during the Le Fort I osteotomy. After removal of the dysplastic bone on the maxilla and debulking at the zygomaticomaxillary region, rigid internal fixation was performed with miniplates (2 mm thick). BSSRO was performed to improve mandibular occlusal canting and remove the dysplastic bone. Additional body shaving and genioplasty were performed to improve esthetics. The postoperative recovery was uneventful. The occlusion was stable after 18 months postoperatively, and there was no evidence of recurrence or relapse. At the time of plate removal, 2 years after the initial surgery, the site of maxillary Le Fort I osteotomy was examined and a significant osseous union was noted between the osteotomized segments (Fig. 4). However, slight expansion of the external cortex of the left maxilla was noted, which did not influence facial symmetry. Four years after the initial surgery, the patient did not show further expansion or re-growth of the dysplastic lesion (Fig. 5).

### Literature review

Including our two cases, 15 documented cases of orthognathic surgery for craniofacial FD involved with dentition have been reported. Most patients were young adults (average age 26.6 ± 5.6 years, range 16–35), with female predominance (4 males and 11 females). Among these patients, five were monostotic, five were polyostotic, and another five were not clearly defined. In most cases, the maxillary or mandibular segment had been rigidly fixed with plates and screws, and orthognathic surgery for FD showed stable results after Le Fort I or BSSRO (Table 1).

**Table 1:** Reported orthognathic surgery for fibrous dysplasia involved with dentition

Case	Reference	Age/sex	Lesion type/site	Surgical procedure/method of fixation	Bone healing/recurrence/stability of occlusion	Follow-up
1	Sachs et al. [5]	18/F	Polyostotic/Rt. maxilla, zygoma, frontal, temporal, sphenoid, rib	Le Fort I and both subcondylar osteotomies/interosseous wiring and maxillomandibular fixation (8 weeks)	Favorable/no/stable	30 months
2	Samman et al. [6]	28/M	Polyostotic/Rt. maxilla, zygoma	Le Fort I/RF (miniplates)	Favorable/no/stable	30 months
3	Samman et al. [6]	24/M	Monostotic/Rt. mandible (body and ramus)	Mandibular step osteotomy/RF with miniplates	Favorable/no/stable	2 years
4	Cheung et al. [7]	32/M	Unspecified/Rt. maxilla, zygoma, mandible	Maxillary segmental and mandibular subapical osteotomy/RF with miniplates	Favorable/no/stable	26 months
5		22/F	Unspecified/Rt. maxilla, zygoma,	Le Fort I, BSSRO, genioplasty/RF with miniplates	Favorable/no/stable	9 months
6		30/F	Monostotic/Lt. mandible (body and ramus)	Maxilla and mandibular segmental osteotomy, genioplasty	Favorable/no/stable	13 months
7		25/F	Monostotic/Rt. mandible (body and ramus)	Mandibular body step osteotomy	Favorable/no/stable	32 months
8	Yeow and Chen (1999)[8]	27/F	Unspecified/Lt. maxilla, zygoma, skull base	Le Fort I (maxillary ridge resection)/RF with miniplates	Favorable/no/stable	1~ 9 years
9		35/F	Unspecified/Lt. fronto-orbito-zygomatico-maxilla	3 piece Le Fort I (left maxillary intrusion)/RF with miniplates	Favorable/no/stable	
10		31/F	Unspecified/Lt. fronto-orbito-zygomatico-maxilla	Le Fort I (left maxillary intrusion)/RF with miniplates	Favorable/no/stable	
11		31/F	Monostotic/Rt. mandible (body and ramus)	BSSRO, genioplasty, body shaving	Favorable/no/stable	
12	Matsuo et al. [9]	29/F	Polyostotic/Rt. maxilla, zygoma, mandible	Le Fort I (navigation surgery) and BSSRO/RF with miniplates	Favorable/no/stable	2 years
13	Magraw et al. [10]	16/M	Polyostotic/Rt. zygoma, maxilla, mandible	BSSRO, genioplasty/RF	Favorable/no/stable	6 months
14	Current report	20/F	Monostotic/Lt. maxilla, zygoma	Le Fort I canting correction, surgical contouring, BSSRO, Mandibular wing osteotomy/RF with miniplates	Favorable/mild bone expansion/stable	2 years
15		31/F	Polyostotic/Lt. naso-ethmoid, maxilla, mandible, zygoma	Le Fort I canting correction, surgical contouring, BSSRO, genioplasty/RF with miniplates	Favorable/mild bone expansion/stable	4 years

*BSSRO* bilateral sagittal split ramus osteotomy, *RF* rigid fixation

## Discussion

One important concern in orthognathic surgery for patients with FD is the long-term stability of the osteotomized segments as FD-involved bones are affected by a dysplastic process and are typically soft and friable [12]. Therefore, it is difficult to tightly fix the screws and miniplates to the fibrodysplastic bone. However, long-term stability of the osteotomized segments and occlusion was achieved in both our cases and the previously presented reports.

Notably, in cases of lower extremity fracture involved with FD, screw fixation is strongly discouraged. When screws are inserted into the FD-involved bone, the procedure is recommended to be used carefully, and only in patients with adequate strength of cortical bone [2]. However, our experience and those of others have noted that the dense residual bone does not usually remain at the FD-involved bones, such as zygomaticomaxillary buttresses, where conventional rigid fixation cannot be achieved [6–8]. Even under these unfavorable conditions, the successful long-term stability after plate/screw fixation of FD-involved osseous segments can be explained. Yeow and Chen [8] suggested that FD of the craniofacial region tends to be more osseous in nature than FD of long bones. Another histological study showed that the dysplastic bone typically healed favorably around the biocompatible titanium screws. Osteointegration was observed between the screws and dysplastic bone [7]. CT imaging of patient no. 1 (Fig. 2) also showed that the interface of the osteotomized bone was healed with dysplastic bone, and the maxillary sinus was obliterated again with a fibrodysplastic lesion. Since the miniplates were covered by the newly formed dysplastic bone, these findings suggest that the dysplastic bone can contribute to the stability of the osseous segments. Because of poor bone quality, it can be challenging, but it is not impossible to achieve adequate fixation on the dysplastic bone intraoperatively, and long-term stability can be expected after the orthognathic surgery. In addition, since FD is not usually bilateral, the normal contralateral side can provide adequate stability if the previous lesion can be healed with softer bone.

Another major concern after FD treatment is recurrence. Depending on the site and extent of involvement, the rate of growth, clinical behavior of the lesion, esthetic disturbance, functional disruption, general health of the patient, and type of surgical intervention can be considered [8, 13]. The prognosis of the monostotic form is reported to be good, whereas prognosis of the polyostotic form is considered to be proportional to the extent of the disease [1]. There is a report that showed that the surgical manipulation can accelerate the re-growth of the remaining FD lesion [14].

In the previously reported 13 cases of orthognathic surgery for FD, no recurrence was reported (Table 1) regardless of monostotic or polyostotic FD. Boyce et al. [15] reported that in patients with craniofacial FD, re-growth and reoperation are more frequent, particularly after debulking procedures, than aggressive reconstructive measures. It has also been suggested that growth hormone excess should be treated prior to surgery to reduce the rate of recurrence after surgery [2, 15]. In some reports, evaluation of biochemical markers, such as serum osteocalcin, and total and bone-specific alkaline phosphatases, has been advised to follow the disease progression [16, 17]. Therefore, the favorable results of our report and previous findings may be explained by the fact that most patients were not syndromic and did not present with endocrine disorders. Another factor is that FD resection and recontouring were performed at the same time during the orthognathic surgery, which is a more aggressive approach than debulking. Since the definition of suitable predictors of the recurrence of FD remains controversial, close follow-up in the long term is emphasized [17, 18].

It is has also been suggested that (1) surgical treatment after confirmation of skeletal maturity and (2) absence of further growth of the dysplastic bone are the most important factors in the successful management of FD affecting the occlusion [3, 4].

While the previously reported cases showed no recurrence of FD, it is unclear whether all cases were examined with CT or the operated site was directly inspected. Although the amount of newly formed bone did not influence facial appearance, we found that dysplastic bone can grow over the miniplates during the healing process and show mild expansion of the lesion.

## Conclusion

Patients with FD of the craniomaxillofacial region often benefit from orthognathic surgery, which may be necessary in cases with higher rates of facial asymmetry and malocclusion. The major concerns in orthognathic surgery for patients with FD are bone reunion, stability, quality of the newly formed bone, and recurrence and relapse following the osteotomy. In the presented cases, we were able to improve facial deformities and functional disturbances after orthognathic surgery. Bone healing was also favorable, similar to other reports. However, we experienced a case of mild growth of dysplastic bone over the osteotomized segments and miniplates, even though it did not significantly affect facial appearance. Therefore, long-term follow-up and monitoring are needed, even in cases where the osteotomized segment shows stable results.

## Abbreviations

FD: Fibrous dysplasia
CT: Computed tomography
